# Comprehensive Genomic Characterization of 102 Cervical Adenocarcinoma Tumors

**DOI:** 10.3390/medicina62010123

**Published:** 2026-01-07

**Authors:** Gejla Toromani, Grace S. Saglimbeni, Bhanu Surabi Upadhyayula, Eugene Manu, Tyson J. Morris, Beau Hsia, Abubakar Tauseef

**Affiliations:** 1 College of Arts and Science, Miami University, Oxford, OH 45056, USA; 2 School of Medicine, Creighton University, Phoenix, AZ 85012, USA; gracesaglimbeni@creighton.edu (G.S.S.); tysonmorris@creighton.edu (T.J.M.);; 3 School of Biological Sciences, University of California, Davis, CA 95616, USA; bupadhyayula@formerstudents.ucdavis.edu; 4 School of Natural Sciences and Mathematics, Hofstra University, Hempstead, NY 11549, USA; eugenemanu4@gmail.com; 5 School of Medicine, Creighton University, Omaha, NE 68178, USA; abubakartauseef@creighton.edu

**Keywords:** cervical adenocarcinoma, genomic profiling, somatic mutations, copy number alterations, *TP53*, *PIK3CA*, *KRAS*, HPV-independent tumors, tumor heterogeneity, precision oncology

## Abstract

***Background and Objectives:*** Cervical adenocarcinoma (CAC) is a histologically distinct subtype of cervical cancer with a rising incidence in many regions. While the roles of key driver mutations are known, a comprehensive understanding of its genomic landscape, particularly variations across different populations and tumor stages, remains incomplete. This study aims to characterize the somatic genomic landscape of CAC by identifying recurrent mutations, copy number alterations (CNAs), and patterns of co-occurrence, with a focus on variations across racial groups and between primary and metastatic tumors. ***Materials and Methods:*** We conducted a comprehensive genomic analysis of 102 tumor samples from 99 patients diagnosed with cervical adenocarcinoma using data from the American Association for Cancer Research (AACR) Project Genomics Evidence Neoplasia Information Exchange (GENIE) database. ***Results:*** The most frequently mutated genes were *PIK3CA* (25.5%), *TP53* (21.6%), *ARID1A* (20.6%), and *KRAS* (16.7%). Significant amplification of *ERBB2* was also observed *(n* = 3; 4.83%). Our analysis revealed notable genomic disparities across racial groups, with *TP53* mutations being significantly more frequent in White patients compared to Asian and Black patients (*p* = 0.0236). Furthermore, we identified significant co-occurrence between mutations in *KRAS* and *MSH2* (*p* = 0.011) as well as *ATM* and *STK11* (*p* = 0.037). In comparing tumor types, mutations in *BCL6* were found to be significantly enriched in metastatic samples. ***Conclusions:*** This study validates the primary drivers of cervical adenocarcinoma and reveals novel findings, including notable racial disparities in *TP53* mutation frequency and unique patterns of co-occurring mutations. These findings highlight the genomic heterogeneity of the disease and suggest that ancestry and tumor evolution may influence its molecular pathogenesis, offering potential avenues for the development of targeted therapies and personalized biomarkers.

## 1. Introduction

Cervical adenocarcinoma (CAC) is a distinct malignant tumor derived from the glandular epithelial cells of the endocervix, representing a growing component of gynecological malignancies [1,2]. While Squamous Cell Carcinoma (SCC) remains the predominant type, CAC accounts for approximately 10% to 25% of all cervical cancers [1]. Despite a global decline in the overall incidence of cervical cancer, the relative and absolute incidence rates of CAC have shown a notable increase in recent decades [3,4]. Incidence rates of cervical cancer show significant geographic and socioeconomic disparities, with the highest rates found in Sub-Saharan Africa, Central America, and Southeast Asia [5]. Globally, the burden of disease is starkly unequal; incidence declines as the Human Development Index (HDI) increases [1]. Notably, low Human Development Index (HDI) countries have incidence rates two times higher and mortality rates five times higher than very high HDI countries [6,7]. The disease can affect a wide age range of women, with CAC tending to affect younger individuals and showing an increasing trend among women in their 20s and 30s [1,7,8].

CAC is recognized as a biologically aggressive entity, distinct from SCC. Adenocarcinoma histology is an independent factor for poorer disease-free survival (DFS) and overall survival (OS) compared with SCC [7]. Multivariate analysis confirms that CAC histology is a significant independent factor for poor OS (Hazard Ratio [HR], 2.32; *p* < 0.0001) [7,9]. This poorer prognosis is exacerbated by the tendency of CAC toward distant recurrence resulting from hematogenous spread (vs. locoregional recurrence common in SCC) [7,8,9]. Established risk factors for cervical cancer include HPV infection, smoking, a weakened immune system (such as from HIV), obesity, and the use of hormonal contraceptives [10,11,12,13]. Specifically, HPV is the primary etiological driver, responsible for up to 92% of all cervical cancers [14,15], with at least 80% of CAC cases being associated with HPV [15,16,17].

Genomic studies have elucidated a complex mutational landscape in CAC, underscoring the involvement of several key oncogenic pathways. Prominent among these is the PI3K/Akt/mTOR signaling cascade, which is frequently altered and represents a promising target for therapeutic intervention [17,18,19,20]. High rates of missense mutations occur in driver genes such as *PIK3CA* and *KRAS* (each ~30%), with additional alterations in MET and RB1 [20,21]. Broader multigene profiling has revealed mutations in *FAT1*, *HLA-B*, *MTOR*, *KMT2D*, and *ZFHX3*, along with frequent copy-number variations in loci such as *PIK3CA*, *BRCA1*, *BRCA2*, *ATM*, and *TP53* [22,23]. Chromosomal amplification of the 3q24–28 region, present in up to 90% of invasive carcinomas, has been recognized as a critical driver of CAC progression [24,25]. Mutations enriched specifically in adenocarcinoma, such as *ELF3* and *CBFB*, further emphasize subtype-specific oncogenic mechanisms [26,27]. The coexistence of *KRAS* and *PIK3CA* mutations in individual tumors highlights synergistic dysregulation across the PI3K and MAPK pathways and underscores the aggressive biology associated with these concurrent alterations [20]. Together, these findings reveal widespread genomic instability across CAC, including missense mutations, frameshift variants, and copy-number gains/losses, which collectively contribute to tumor initiation, progression, and destructive invasion.

Cervical adenocarcinoma comprises several histological subtypes with distinct etiologies and clinical behaviors. The most common subtype is usual-type (HPV-associated) adenocarcinoma, which accounts for the majority of cases. Less frequent subtypes include gastric-type adenocarcinoma, clear cell adenocarcinoma, and other rare variants, many of which are HPV-independent and associated with more aggressive clinical behavior. Although detailed histopathologic subclassification was not available in the AACR GENIE dataset, the inclusion of biologically diverse adenocarcinoma cases underscores the importance of comprehensive genomic characterization to identify shared, subtype-agnostic molecular drivers that may inform prognosis and therapeutic targeting.

The less common HPV-independent (HPVi) CAC subtypes, such as Clear Cell Adenocarcinoma (CCAC) and Gastric-type Adenocarcinoma (GAC), also possess unique molecular landscapes. CCAC, which has a peak incidence in adolescence, is an aggressive, typically HPV-independent tumor [17,28,29]. Studies of juvenile CCAC cases revealed genetic alterations in unique genes such as *ALKBH7*, *MYCBP*, *MZF1*, *RNF207*, *RRS1*, and *TUSC2*, which are infrequently researched in the context of cervical cancer [28]. Furthermore, genomic and single cell sequencing studies emphasize that CAC generally exhibits a weaker immune infiltration and an inactive Tumor Microenvironment (TME) compared to SCC [30,31]. This difference is particularly pronounced in non-HPV cervical cancers, where downregulated genes are primarily related to the immune system response, consistent with the weak link to viral infection [27].

The prognostic value of these mutations is reflected in clinical outcomes, particularly across different demographic groups. For women living with HIV (WLH), the incidence of CAC is modestly elevated (Standardized Incidence Ratio [SIR] 1.47) compared to the general population, though still lower than the increase observed for SCC (SIR 3.62) [12,32,33]. However, among WLH, mortality from CAC remains significantly higher than in women not living with HIV (HR 2.52) [12,32]. Collectively, these clinical and molecular patterns highlight CAC’s aggressive behavior and the need for integrating genomic and histopathological markers, such as Silva pattern-based classification, into risk assessment and treatment planning [34,35,36,37]. Yet despite these insights, substantial gaps remain in understanding the full genomic architecture of CAC.

Despite recent advances, a comprehensive genomic profile of cervical adenocarcinoma, particularly in specific populations, is lacking. This knowledge gap limits the ability to refine prognostic markers and identify novel therapeutic targets. This study aims to characterize the somatic genomic landscape of cervical adenocarcinoma using the AACR GENIE dataset. The findings from this research have the potential to identify new avenues for targeted therapies and improve risk stratification, ultimately contributing to more personalized and effective management of this cancer subtype.

## 2. Materials and Methods

The data used in this study were accessed from the American Association for Cancer Research (AACR) Project Genomics Evidence Neoplasia Information Exchange (GENIE) database. The study conducted by Creighton University (Phoenix, AZ, USA) was exempt from Institutional Review Board approval as it uses publicly available, de-identified data. The data were collected on 4 June 2025, from the online software cBioPortal (v17.0-public) and consisted of clinical and genomic data from 2017 onwards. The AACR GENIE dataset is composed of genomic sequencing data from 19 international cancer centers. This database combines whole genome sequencing (WGS), whole exome sequencing (WES), and targeted gene panels (spanning 50–555 genes) to demonstrate heterogeneity through the genome sequencing. Overall, 80% of samples underwent targeted panel sequencing, 15% WES, and 5% WGS. Targeted panels achieved >500× coverage, compared with 150× for WES and 40× for WGS.

Each AACR GENIE participating institution follows its own guidelines for mutation calling and annotation. Due to these institutional differences, variability in bioinformatic pipelines may exist. However, all data were standardized through GENIE harmonization protocols via Genome NEXUS, which uses common tools such as GATK for variant detection and ANNOVAR for annotation, although specific software and versions vary by institution. Given the use of multiple sequencing platforms across contributing institutions, including targeted gene panels, whole-exome sequencing, and whole-genome sequencing, some variability in mutation detection sensitivity is unavoidable. Although linear regression models were applied to adjust for differences in sequencing panel size, residual platform-related bias cannot be fully excluded, particularly for low-frequency or subclonal variants. To further clarify, linear regression was applied at the sample level to adjust for heterogeneity in sequencing panel size. However, gene-specific normalization based on panel-level coverage was not performed, genes absent from certain panels were not excluded from analyses on a per-gene basis, and sensitivity analyses restricted to comparable sequencing platforms were not conducted. These factors should be considered when interpreting gene-specific mutation frequencies, as they may introduce residual bias.

Clinical outcome and therapeutic response data were not available for the cervical adenocarcinoma cases included in this study. Detailed clinical outcome and survival data were not uniformly available for cervical adenocarcinoma cases in this cohort; therefore, prognostic analyses correlating specific genomic alterations with clinical outcomes or survival endpoints could not be performed.

Our study cohort consisted of patients with a pathologic diagnosis of cervical adenocarcinoma, drawn from a larger cohort of cervical cancer cases. Tumor samples included both primary tumors (originating at the initial site) and metastatic tumors (obtained from distant disease sites). The dataset included clinical demographics (age, sex, race, etc.), histological classification, and genomic data. Missing or insignificant data points, non-actionable genes, structural variants, and synonymous mutations were excluded from this study. While targeted sequencing panels differed between institutions, most included frequently mutated cancer-associated genes such as *PIK3CA*, *TP53*, and *ARID1A*. Mutations included in the analysis were missense, nonsense, frameshift, and splice-site variants with reported variant allele frequencies. Mutation calls were obtained from GENIE harmonized mutation annotation format files, which provide standardized variant annotations across all institutions. Copy number alterations (CNAs), including homozygous deletions and amplifications, were evaluated for their frequency. Both primary and metastatic tumor samples were included to maximize available genomic data in this rare malignancy. Given the potential for genomic divergence between primary and metastatic lesions, subgroup analyses were performed to compare mutation frequencies between these groups. Findings from primary-metastatic comparisons are interpreted cautiously and are considered exploratory. Tumor samples were categorized based on AACR GENIE annotations, including primary tumors, distant organ metastases, lymph node metastases, and local recurrences. For analyses comparing primary versus metastatic tumors, all non-primary categories were grouped together as metastatic samples, while primary tumors were analyzed separately. Sample counts for each category are consistently reported across all analyses.

Continuous variables are reported as mean ± standard deviation (SD) with normality assessed. Normally distributed variables were compared using a two-sided Student’s *t*-test, while non-normal variables were compared using the Mann–Whitney U test. Categorical variables are reported as frequencies and percentages, with group comparisons made using the chi-squared test. Race-stratified comparisons were conducted using chi-squared tests; Fisher’s exact test was not applied, and no correction for multiple testing was performed for these subgroup analyses. These limitations should be considered when interpreting race-specific findings. Co-occurrence analyses were conducted to identify statistically significant associations between recurrently mutated genes. Due to the modest cohort size and the large number of gene-pair comparisons, multiple testing correction was not applied. Therefore, these findings are considered exploratory and hypothesis-generating rather than definitive.

Linear regression models were applied to adjust for panel size variation, enabling comparability across sequencing platforms. Statistical analyses were performed in R/RStudio (R Foundation for Statistical Computing, Boston, MA, USA, version 4.5.1). A *p*-value < 0.05 was considered statistically significant, with the Benjamini–Hochberg false discovery rate (FDR) correction applied to adjust for multiple comparisons. 

## 3. Results

### 3.1. Patient Demographics of Cervical Adenocarcinoma

The demographic analysis combined various tumor samples of cervical adenocarcinoma amongst a limited sample size. The demographics are detailed in Table 1 and include data from 99 patients and 102 samples. Amongst these patients, 98 (99.0%) were female and 1 (1.0%) was unknown. By ethnicity, the majority were non-Hispanic 60 (60.6%), followed by unknown/not collected 27 (27.3%) and Hispanic 12 (12.1%). In terms of race, the cohort included White 64 (64.6%), unknown 12 (12.0%), Black 7(7.1%), Asian 6 (6.1%), and not collected 5 (5.1%). Most patients (n = 97; 95%) were over the age of 30, while 5 (4.9%) were below the age of 30. Tumor types consisted of primary (n = 54; 52.9%), metastasis (n = 25; 24.5%), not specified (n = 7; 6.9%), distant organ (n = 6; 5.9%), not collected (n = 4; 3.9%), lymph node metastasis (n = 3; 2.9%), local recurrence (n = 2; 2.0%), and not applicable (n = 1; 1.0%). 

### 3.2. Most Common Somatic Mutations and Copy Number Alterations

**Figure 1:**
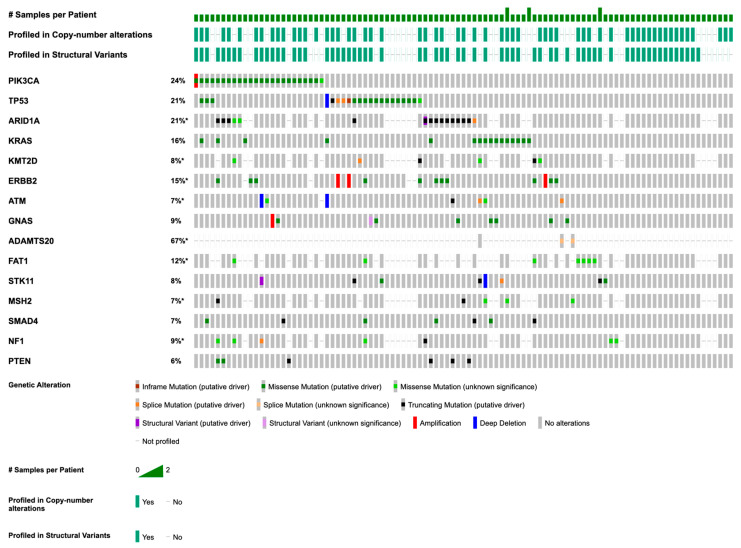
OncoPrint of recurrent somatic mutations and copy number alterations in cervical adenocarcinoma. This OncoPrint displays recurrent somatic mutations and copy number alterations (CNAs) across 102 cervical adenocarcinoma tumor samples from 99 patients. Mutations include missense, nonsense, frameshift, and splice-site variants. CNAs include amplifications (AMP) and homozygous deletions (Loss). Rows represent genes, and columns represent tumor samples. Gene frequency and mutation type are annotated by color. Genes marked with an asterisk (*) are significantly mutated genes identified by statistical analysis, indicating mutation frequencies higher than expected by chance. The hashtag symbol (#) indicates the number of samples analyzed per patient.

The most common mutations were in *PIK3CA* (n = 26; 25.5%), *TP53* (n = 22; 21.6%), *ARID1A* (n = 21; 20.6%), *KRAS* (n = 17; 16.6%), *KMT2D* (n = 12; 11.8%), *ERBB2* (n = 11; 10.8%) *ATM* (n = 8; 7.8%), *GNAS* (n = 8; 7.8%), *ADAMTS20* (n = 8; 7.8%), *FAT1* (n = 7; 6.9%), *STK11* (n = 7; 6.9%), *MSH2* (n = 7; 6.9%), *SMAD4* (n = 7; 6.9%), *NF1* (n = 7; 6.9%), and *PTEN* (n = 7; 6.9%) (Table 2; Figure 1).

Among the 62 samples profiled for copy number alterations (CNAs), amplification (AMP) events were highly prevalent in *ERBB2*, *CDK12*, *MYC* (n = 3; 4.8% for all) (Figure 1). There were also amplification events seen in *SOC2*, *PRKCI*, *EIF4A2* (n = 2; 3.2% for all). A loss of function event was detected in *ATM* (n = 2; 3.2%) (Table 3; Figure 1).

In reporting recurrent alterations, well-established oncogenic drivers, including *PIK3CA*, *TP53*, *ARID1A*, and *KRAS*, were emphasized in downstream interpretation. Additional low-frequency or less-characterized variants are presented for descriptive completeness but may represent passenger events rather than functionally relevant drivers.

### 3.3. Genetic Differences by Sex and Race

**Figure 2:**
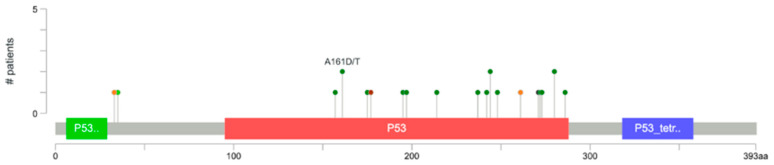
Somatic mutation hotspots across recurrently mutated genes in cervical adenocarcinoma. Hotspot mutations are shown for recurrently mutated genes (*PIK3CA*, *TP53*, *ARID1A*, *KRAS*, *KMT2D*, *ERBB2*, *ATM*, *GNAS*, *ADAMTS20*, *FAT1*, *STK11*, *MSH2*, *SMAD4*, *NF1*, *PTEN*) across 102 tumor samples from 99 patients. Mutation types are color-coded: missense, nonsense, frameshift, and splice-site variants. Sample size (n) per mutation is annotated. Colored boxes represent p53 protein domains, including the N-terminal region (‘P53..’, green), the DNA-binding domain (‘P53’, red), and the C-terminal tetramerization domain (‘P53_tetr..’, blue). The y-axis (#) indicates the number of patients. This figure highlights the distribution and frequency of recurrent somatic mutations.

When stratified by sex, no statistically significant findings were observed, as the study consisted of 99% female patients and *p*-values were >0.05. Race-based comparisons were conducted among Asian, Black and White patients. Genes demonstrating statistically significant differences in mutation frequency across racial groups are summarized in Table 4. Through this cohort, many mutations could be identified as statistically significant with *p* values below 0.05. The gene with the most samples of mutations with significant values was *TP53*, significantly enriched in White patients in comparison to Asian and Black patients (n = 12 vs. n = 4 vs. n = 2; *p* = 0.0236) (Figure 2).

### 3.4. Co-Occurrence and Mutual Exclusivity of Mutations

There were no significant mutual exclusivity patterns observed. However, significant co-occurrence was seen between *KRAS* and *MSH2* (*p* = 0.011) as well as *ATM* and *STK11* (*p* = 0.037).

**Table 4:** Race-stratified distribution of significantly mutated genes in cervical adenocarcinoma. Distribution of recurrent somatic mutations stratified by race (Asian, Black, White) among 102 tumor samples from 99 patients. Only genes with statistically significant differences (*p* < 0.05) are included. Mutations are shown with corresponding counts (only >1 selected) and *p*-values.

Gene	Asian	Black	White	*p*-Value
*MECOM*	1	0	1	*p* < 0.001
*ADAMTS20*	0	0	2	*p* < 0.001
*BOD1L1*	0	0	2	*p* < 0.001
*CACNA1E*	0	0	2	*p* < 0.001
*PKD1L2*	0	0	2	*p* < 0.001
*PKHD1*	0	0	2	*p* < 0.001
*TAF1L*	0	0	2	*p* < 0.001
*TNK2*	0	0	2	*p* < 0.001
*FAAP100*	1	0	2	0.0137
*CA SP8*	1	0	1	0.0151
*SYNE1*	0	0	3	0.0183
*TP53*	4	2	12	0.0236
*DNAH9*	0	0	2	0.0251
*NF2*	1	0	1	0.0313
*ASXL1*	1	0	1	0.0354
*NBN*	1	0	1	0.0354
*TNFAIP3*	1	0	1	0.0354

### 3.5. Primary vs. Metastatic Mutations

This cervical adenocarcinoma study had 4 primary samples and 25 metastasis CAC cases. In the comparative genomic alterations analysis, there were 54 primary and 36 metastases. Notably, significant enrichment was identified in the metastasis samples, which included *BCL6* (n = 4; 0.0216), as well as *CLTCL1* (n = 1; *p* = 0.0357). There were no significant differences detected between the primary and metastatic groups. 

## 4. Discussion

### 4.1. Overview and Key Findings

This study utilized a multi-institutional genomic dataset to characterize the somatic mutational landscape of cervical adenocarcinoma. Our analysis demonstrated a heterogeneous molecular profile, highlighted by recurrent alterations in *PIK3CA*, *TP53*, *ARID1A*, and *KRAS*, as well as gene amplifications in *ERBB2*, *CDK12*, and *MYC*. Notably, significant differences in mutation frequency were observed across racial subgroups, and distinct patterns of co-occurring mutations emerged, underscoring the complex genomic landscape of this malignancy (Table 4) [38,39,40].

### 4.2. Demographic Patterns and Genomic Variation

Our cohort of cervical adenocarcinoma was overwhelmingly comprised of female patients (99%), consistent with the established epidemiology of this malignancy, which almost exclusively affects women due to the anatomical origin of the cervix [7,13,20]. The largest ethnic group in our study was non-Hispanic (60.6%), followed by unknown/not collected (27.3%), and Hispanic (12.1%). The racial composition consisted of White (n = 64, 64.6%), unknown (n = 12, 12.1%), Black (n = 7, 7.1%), Asian (n = 6, 6.1%), and not collected (n = 5, 5.1%). The vast majority of cases were diagnosed in women over 30 years of age (n = 97/102, 95.1%), which aligns with the known age distribution of cervical adenocarcinoma, most commonly diagnosed between 35 and 44 years of age, with an average age at diagnosis of 50 years and rarity in those younger than 20 [4,12,15,28].

When stratified by race, notable molecular differences emerged. Most strikingly, *TP53* mutations were significantly more frequent in White patients than in Asian or Black subgroups (n = 12 vs. n = 4 vs. n = 2, *p* = 0.0236), indicating possible genetic susceptibility or environmental exposures unique to this demographic group (Table 4). While prior studies have described the overall mutational landscape of cervical adenocarcinoma [15,28], specific racial enrichment patterns for key drivers like *TP53* have not been widely reported, making this a novel observation. The mechanisms underlying these disparities remain unclear, though differences in human papillomavirus (HPV) subtypes, host genetic factors, and access to screening and preventive care may be contributing factors [28]. The cohort was overwhelmingly female, reflecting the near-exclusive occurrence of cervical adenocarcinoma in women [15,28]. Similarly, age-specific mutation trends could not be robustly analyzed due to the small number of patients under 30 years old.

Collectively, the observed demographic-specific genomic patterns, especially the enrichment of *TP53* mutations in White patients, suggest underlying etiologic or biological variation that may influence tumor evolution and therapeutic response. These findings underscore the importance of considering ancestry and demographic context in future molecular and clinical studies and highlight the need for continued investigation into population-based differences in cervical adenocarcinoma pathogenesis and outcomes.

Nonetheless, these race-specific enrichments should be interpreted with caution. The statistical power of this subgroup analysis is limited by the small number of patients in the Asian (n = 6) and Black (n = 7) cohorts, as well as by the substantial proportion of cases with unknown or uncollected race information (Table 4). Consequently, these findings are susceptible to statistical instability and may reflect stochastic variation rather than true population-level differences. Moreover, potentially relevant confounding factors, including HPV subtype distribution, environmental exposures, and disparities in access to screening or care, could not be assessed within the constraints of this dataset. Accordingly, these observations should be considered exploratory and require validation in larger, more diverse, and clinically annotated cohorts before definitive conclusions can be drawn. Race-stratified analyses were limited by very small subgroup sizes and the lack of multiple testing correction. As such, statistically significant findings, particularly those driven by single-event mutations, should be interpreted cautiously and regarded as exploratory rather than definitive population-level associations.

### 4.3. Frequently Mutated Genes and Pathways

Our cervical adenocarcinoma cohort exhibited genetic diversity, with frequent mutations in *PIK3CA* (n = 26; 25.5%), *TP53* (n = 22; 21.6%), *ARID1A* (n = 21; 20.6%), *KRAS* (n = 17; 16.7%), and *KMT2D* (n = 12; 11.8%) (Figure 2). Notable recurrent alterations also included *ATM*, *GNAS*, *ADAMTS20*, *FAT1*, *STK11*, *MSH2*, *SMAD4*, *NF1*, and *PTEN*. We observed amplification events in *ERBB2*, *CDK12*, and *MYC* (n = 3; 4.8% each), as well as in *SOC2*, *PRKCI*, and *EIF4A2* (n = 2; 3.2% each).

These findings align with previous genomic studies of cervical adenocarcinoma, which highlight *PIK3CA*, *TP53*, and *KRAS* as key drivers, frequently implicated in the PI3K/AKT, MAPK, and cell cycle regulatory pathways [37,38,39,40]. Mutations in *ARID1A* and *KMT2D* indicate that disrupted chromatin remodeling has also been found to be relevant in tumorigenesis [37,38,39]. Importantly, the identification of *ERBB2* amplification suggests potential for *HER2*-targeted therapies in a subset of patients [39,40].

#### 4.3.1. PI3K/AKT Pathway (PIK3CA)

*PIK3CA* was the most frequently mutated gene in our cervical adenocarcinoma cohort (n = 26, 25.5%), a finding consistent with previous studies showing *PIK3CA* mutation rates of 25–42% in cervical adenocarcinoma and squamous cell carcinoma [37]. These mutations predominantly occur at hotspot sites such as *E542K* and *E545K*, which are well-known to activate the PI3K/AKT pathway, promoting cell growth, survival, and resistance to apoptosis [37,38,39]. Notably, *PIK3CA* mutations often arise as late events in cervical carcinogenesis, marking the transition from preinvasive to invasive disease [39].

Clinically, *PIK3CA* mutations and pathway activation have been linked to higher tumor mutation burden and poorer prognosis, including worse progression-free and overall survival [40]. Importantly, these alterations have therapeutic relevance: several PI3K/AKT/mTOR inhibitors have been investigated in early phase trials for gynecologic tumors, and the PI3Kα inhibitor alpelisib has shown preclinical efficacy against *PIK3CA*-mutant cervical carcinoma models [40]. Detection of *PIK3CA* mutations in circulating tumor DNA may allow for real-time monitoring of disease and tailoring of targeted therapy [37,40].

#### 4.3.2. TP53 Pathway

*TP53* mutations were observed in 21.6% of cases in our cohort, mirroring reported frequencies in Western and Asian cervical adenocarcinoma populations [38]. *TP53* acts as a central tumor suppressor, orchestrating the response to DNA damage and preventing the propagation of genetically unstable cells. Loss-of-function *TP53* mutations confer genomic instability and resistance to apoptotic cues, contributing to tumor aggressiveness [37,38,39,40]. In cervical adenocarcinoma, such mutations are associated with high-grade tumors and worse clinical outcomes [37,38,39,40].

Currently, direct targeting of mutant *TP53* remains a challenge, but several therapeutic approaches are under investigation, including reactivators of mutant *p53* protein and strategies exploiting synthetic lethality with agents inducing DNA damage. Recent data also suggest that *TP53* status may impact response to chemotherapy and emerging immunotherapies [39,40].

#### 4.3.3. MAPK Pathway (KRAS)

*KRAS* mutations were detected in 16.7% of cases, consistent with the literature reporting *KRAS* as a recurrent alteration in 10–20% of cervical adenocarcinomas, particularly in HPV-negative and gastric-type tumors [39,40]. Mutant *KRAS* chronically activates the *MAPK* pathway, driving growth and inhibiting apoptosis. While direct targeting of *KRAS* has proven difficult, *KRAS G12C* inhibitors are now being explored clinically in other solid tumors. Furthermore, upstream/downstream pathway inhibitors (e.g., *MEK* inhibitors) are under investigation for *KRAS*-driven gynecologic cancers [37,38].

Several recurrent alterations identified in this cohort, including activating mutations in *PIK3CA* and cervical adenocarcinoma, comprise several histological subtypes with distinct etiologies and clinical behaviors. The most common subtype is usual-type (HPV-associated) adenocarcinoma, which accounts for the majority of cases. Less frequent subtypes include gastric-type adenocarcinoma, clear cell adenocarcinoma, and other rare variants, many of which are HPV-independent and associated with more aggressive clinical behavior. Although detailed histopathologic subclassification was not available in the AACR GENIE dataset, the inclusion of biologically diverse adenocarcinoma cases underscores the importance of comprehensive genomic characterization to identify shared, subtype-agnostic molecular drivers that may inform prognosis and therapeutic targeting amplifications of *ERBB2*, which have potential therapeutic relevance based on prior studies in gynecologic malignancies. However, these genomic findings should not be interpreted as immediately clinically actionable in cervical adenocarcinoma. Rather, they identify candidate pathways for future investigation and underscore the potential role of comprehensive genomic profiling in informing personalized therapeutic strategies, pending prospective clinical validation.

### 4.4. Co-Occurrence and Mutual Exclusivity

In our cervical adenocarcinoma cohort, significant co-occurrence was observed between *KRAS* and *MSH2* (*p* = 0.011) and between *ATM* and *STK11* (*p* = 0.037) (Figure 1). This suggests possible cooperative roles in tumor development, such as combined effects on growth signaling (*KRAS*) with DNA repair deficiency (*MSH2* or *ATM*), a relationship not widely reported in prior cervical cancer studies [37,40]. No significant mutual exclusivity was observed among common drivers like *PIK3CA* and *TP53*, consistent with existing literature indicating frequent overlapping mutations in this cancer type [38,39,40]. These co-occurrence patterns may reflect pathway synergy in tumorigenesis and highlight the potential need for combinations of targeted therapies in cervical adenocarcinoma. Although the observed co-occurrence of *KRAS* with *MSH2* and *ATM* with *STK11* is biologically plausible, functional validation of these relationships was beyond the scope of this study. Consequently, these associations should be interpreted as hypothesis-generating rather than as evidence of definitive mechanistic interactions. Because co-occurrence analyses involved multiple gene-pair comparisons without correction for multiple testing, the possibility of false-positive associations cannot be excluded. Therefore, the observed co-occurrence patterns should be considered exploratory and hypothesis-generating, and they require validation in independent cohorts.

### 4.5. Comparison of Primary and Metastatic Tumors

When comparing the 54 primary and 36 metastatic cervical adenocarcinoma samples in our study, *BCL6* and *CLTCL1* mutations were found exclusively in metastatic tumors. *BCL6* was present in 11.1% of metastatic tumors (n = 4), while *CLTCL1* alterations appeared in one metastatic sample (2.8%).

The enrichment of *BCL6* mutations in metastatic samples, though based on small numbers, suggests a potential link to advanced tumor progression. While *BCL6* has been most widely studied in hematologic malignancies, some solid tumor data, such as hypermethylation of *BCL6B* correlating with metastasis in hepatocellular carcinoma, point to its involvement in metastatic behavior [40]. Our findings generally match prior reports indicating that the genomic landscapes of primary and metastatic cervical adenocarcinoma are largely similar, with key driver genes (*PIK3CA*, *TP53*, *KRAS*) distributed evenly [40]. However, unique alterations such as *BCL6* in metastatic lesions may represent late events or adaptations important for tumor spread and therapy resistance. These preliminary observations highlight the need for further validation in larger cohorts but suggest that targeting such alterations could be relevant in the context of metastatic disease. Several observations in this study are based on very small numbers of events, including *ERBB2* amplification and the enrichment of *BCL6* mutations in metastatic samples. While these findings are intriguing, they should be considered exploratory and hypothesis-generating rather than definitive evidence of biological significance.

### 4.6. Study Limitations

While this study offers valuable insight into the genomic features of cervical adenocarcinoma, several limitations should be acknowledged. First, this investigation was reliant on a multi-institutional database that does not provide transcriptome, microRNA, or DNA methylation data, preventing exploration of whether the observed mutations translate to altered gene expression, pathway activation, or epigenetic regulation. As a result, we were unable to link genetic findings to transcriptional or regulatory consequences, missing potentially relevant associations with tumor physiology and therapeutic targets. Second, detailed clinical and therapeutic data, including treatment regimens, response to therapy, and survival outcomes, were not uniformly available in the dataset. The absence of comprehensive clinical outcome and survival information precluded the assessment of prognostic associations between specific genomic alterations and patient outcomes, limiting our ability to correlate molecular findings with disease-free or overall survival.

Third, the modest cohort size, particularly upon subgroup stratification (e.g., by race or metastatic status), diminishes statistical power, making it challenging to establish robust associations between rare mutations and clinical or pathologic features. Larger, more uniformly annotated cohorts will be vital for confirming these preliminary observations. Fourth, the data analyzed were compiled from various institutions and sequencing platforms, potentially introducing batch effects or technical variability that could affect mutation detection rates, copy number call accuracy, or other genomic measurements. Fifth, the cross-sectional design and lack of longitudinal sampling prevented the assessment of genetic evolution or clonal selection over time. Thus, relationships between mutations found in primary versus metastatic tumors, or before and after therapy, could not be explored. The inclusion of metastatic tumor samples may introduce bias, as metastatic lesions can acquire additional genomic alterations during disease progression and clonal evolution. Accordingly, comparisons between primary and metastatic tumors in this study should be regarded as hypothesis-generating and require validation in larger cohorts with longitudinal sampling. Sixth, we were unable to distinguish driver mutations from incidental passenger alterations, given the limited number of samples and the high underlying tumor mutational burden typical of some cervical cancers. Seventh, the study’s dataset did not include immunohistochemical or proteomic data, restricting our ability to evaluate the downstream protein-level impact of genomic alterations or to relate findings to tumor immune microenvironment features. Eighth, all major subtypes of cervical adenocarcinoma were grouped for this analysis, as detailed histopathologic subclassification was unavailable. This limitation made it impossible to identify subtype-specific mutational landscapes or examine whether certain genetic aberrations are unique to, for example, gastric-type or HPV-independent adenocarcinoma.

Despite these limitations, our integrated genomic analysis provides valuable insight into recurrent alterations in cervical adenocarcinoma, highlighting important targets for future translational and clinical research. Taken together, while this study identifies recurrent genomic alterations, it remains primarily descriptive and exploratory. The study design reflects the inherent challenges of investigating a rare malignancy using multi-institutional genomic datasets. Although the inclusion of heterogeneous sample types, such as primary and metastatic tumors from multiple institutions, introduces potential variability, it allows for the identification of recurrent genomic alterations and generates hypotheses for future investigation. Confirmatory conclusions and translation to clinical practice will require validation in larger, uniformly sequenced cohorts with comprehensive clinical and outcome data.

## 5. Conclusions

Going forward, research should focus on validating these findings with integrated clinical outcomes, therapeutic data, and comprehensive multi-omic profiling. Functional studies are needed to clarify the biological and therapeutic relevance of under-characterized genes like *BCL6* and *CLTCL1*, particularly in the context of metastatic progression. Additionally, efforts to subtype cervical adenocarcinoma more precisely and to correlate molecular profiles with treatment response will be essential for individualized patient care. Ultimately, these steps will advance our understanding of cervical adenocarcinoma biology and support the development of personalized therapies to improve outcomes for patients facing this challenging disease [31,35,36].

## Figures and Tables

**Table 1:** Patient demographics of cervical adenocarcinoma cases. Demographic characteristics of 99 patients with cervical adenocarcinoma (102 tumor samples). Sex, age, ethnicity, race, and tumor sample type are shown. Most patients were female (n = 98; 99.0%) and over 30 years of age (n = 97; 95.1%). Sample types include primary tumors (n = 54), metastases (n = 25), and other categories (n = 23).

Demographics	Category	N (%)
Sex	Male	0 (0.0%)
	Female	98 (99.0%)
	Unknown	1 (1.0%)
Age category	Adult (>30)	97 (95.1%)
	Adult (<30)	5 (4.9%)
Ethnicity	Non-Hispanic	60 (60.6%)
	Unknown/Not Collected	27 (27.3%)
	Hispanic	12 (12.1%)
Race	White	64 (64.6%)
	Unknown	12 (12.1%)
	Black	7 (7.1%)
	Asian	6 (6.1%)
	Not Collected	5 (5.1%)
Sample Type	Primary	54 (52.9%)
	Metastasis	25 (24.5%)
	Unknown	16 (15.7%)

**Table 2:** Frequency of recurrent somatic mutations in cervical adenocarcinoma tumors. Recurrent somatic mutations across 102 tumor samples from 99 patients. Mutation types included missense, nonsense, frameshift, and splice-site variants.

Gene	N	Frequency
*PIK3CA*	26	25.5%
*TP53*	22	21.6%
*ARID1A*	21	20.6%
*KRAS*	17	16.6%
*KMT2D*	12	11.8%
*ERBB2*	11	10.8%
*ATM*	8	7.8%
*GNAS*	8	7.8%
*ADAMTS20*	8	7.8%
*FAT1*	7	6.9%
*STK11*	7	6.9%
*MSH2*	7	6.9%
*SMAD4*	7	6.9%
*NF1*	7	6.9%
*PTEN*	7	6.9%

**Table 3:** Recurrent copy number alterations detected in cervical adenocarcinoma tumors. Copy number alterations (CNAs) across 62 tumor samples. CNAs include amplifications (AMP) and loss/homozygous deletion (Loss). Genes with recurrent CNAs include *ERBB2*, *CDK12*, *MYC*, *SOX2*, *PRKCI*, *EIF4A2*, and *ATM*. Frequencies (%) are indicated for each gene.

Gene	N	Frequency	Loss/Amplification
*ERBB2*	3	4.8%	AMP
*CDK12*	3	4.8%	AMP
*MYC*	3	4.8%	AMP
*SOX2*	2	3.2%	AMP
*PRKCI*	2	3.2%	AMP
*EIF4A2*	2	3.2%	AMP
*ATM*	2	3.2%	Loss

## Data Availability

The data presented in this study are available from the AACR GENIE Database at https://genie.cbioportal.org/ (accessed on 4 June 2025).

## Abbreviations

The following abbreviations are used in this manuscript:

AACR	American Association for Cancer Research
AMP	Amplification
CAC	Cervical adenocarcinoma
CCAC	Clear cell adenocarcinoma of the cervix
CNA(s)	Copy number alteration(s)
DFS	Disease-free survival
FDR	False discovery rate
GAC	Gastric-type adenocarcinoma
GENIE	Genomics Evidence Neoplasia Information Exchange
HDI	Human Development Index
HIV	Human immunodeficiency virus
HPV	Human papillomavirus
HPVi	HPV-independent
HR	Hazard ratio
MAPK	Mitogen-activated protein kinas
mTOR	Mechanistic/mammalian target of rapamycin
OS	Overall survival
PI3K	Phosphatidylinositol 3-kinase
SCC	Squamous cell carcinoma
SD	Standard deviation
SIR	Standardized incidence ratio
TME	Tumor microenvironment
WES	Whole exome sequencing
WGS	Whole genome sequencing
WLH	Women living with HIV
